# Nut consumption and risk of diabetes mellitus in overweight/obese individuals

**Published:** 2020-05-29

**Authors:** Omid Eslami, Farzad Shidfar

**Affiliations:** 1 Department of Nutrition, School of Public Health, Iran University of Medical Sciences, Tehran, Iran

## ⁯⁯⁯⁯

***Dear Editor,***

Diets rich in energy-dense foods have generally been associated with an increased risk of obesity and related metabolic disorders (Perez-Escamilla et al., 2012[8]). However, accumulating evidence suggests that nuts, in contrary to the other energy-dense foods, might exert a protective effect against metabolic disorders (de Souza et al., 2017[2]). This might be partly due to the fact that they are also a nutrient-dense source comprising various cardio-protective compounds particularly plant protein, fiber, unsaturated fatty acids, vitamins and minerals (Eslami et al., 2019[3]).

In a recent paper written by Mohammadifard and colleagues (2019[7]) the long-term effect of nut consumption on cardiometabolic risk factors was investigated. The study was comprised of a population with a diverse socio-demographic background from a developing country and specifically a non-Mediterranean region that had followed-up for 12 years; therefore, it provides valuable clues about the health outcomes of nut consumption in the long term. Overall, the authors reported that intake of nuts was inversely associated with risk of general obesity, which supports the findings of our recent review of 6 prospective cohort studies, indicating a protective effect of nut intake against weight gain and risk of overweight/obesity (Eslami et al., 2019[3]). 

Another remarkable finding of this study was that in overweight and obese subjects (i.e. those with a body mass index (BMI) ≥ 25 kg/m^2^), a higher intake of nuts was positively associated with risk of diabetes mellitus (DM). Regarding that individuals with elevated BMI are an at-risk group for the development of insulin resistance and DM, the authors recommended the lower consumption of nuts in this group. However, we believe that this finding should be interpreted in light of the background diet of the study population as well as the type of nuts consumed. Nuts are a major contributor to dietary energy density, which has been linked to the risk of obesity and DM (Hingle et al., 2017[5]; Rouhani et al., 2016[9]). However, evidence from some randomized controlled trials (RCTs) has shown no adverse changes in body weight or glycemic status when they consumed in the context of a healthy dietary pattern such as the Mediterranean diet (Estruch et al., 2019[4]; Lasa et al., 2014[6]). Therefore, the background diet of the study population might modify the health outcomes of energy-dense nuts in the long term. Furthermore, in this study, total nut consumption was considered as the overall intake of almonds, pistachios, hazelnuts, and walnuts. While data on the frequency of consumption of other types of nuts particularly peanuts and cashews were not collected. Notably, in a recent RCT by Abbaspour et al. (2019[1]), consumption of 42.5 g/day mixed-nuts including almonds, walnuts, pistachios, peanuts, hazelnuts, cashews, pecans, Brazil nuts, and macadamia nuts, for 8 weeks was accompanied by a significant decrease in glycemic markers in overweight/obese adults. Since the different types of nuts represent a considerable variation in terms of energy content, fatty acids ratios, micronutrients, and bioactive compounds (Souza et al., 2015[10]), it is possible that this finding might be affected by the classification of nuts in the study. 

Nevertheless, these factors should be taken into account when judging whether the benefits of nut intake on metabolic health outweigh its downsides. Meanwhile, we recommend that more studies are needed in metabolically at-risk subjects, particularly overweight/obese subjects to provide rigorous evidence on this topic.

## Conflict of interest

The authors declare that they have no conflict of interest.

## Financial support

This research did not receive any specific grant from funding agencies in the public, commercial, or not-for-profit sectors.
